# Hepatic paragonimiasis in a 15-month-old girl: a case report

**DOI:** 10.1186/s12887-017-0942-5

**Published:** 2017-11-15

**Authors:** Zongrong Gong, Zhicheng Xu, Chuanfen Lei, Chaomin Wan

**Affiliations:** 1 Department of Pediatrics, West China Second University Hospital, Sichuan University, No. 20,3rd Section of Renmin South Road, Chengdu, 610041 China; 2 Key Laboratory of Birth Defects and Related Diseases of Women and Children, Ministry of Education, Sichuan University, Chengdu, Sichuan China; 3 Department of Pediatric Surgery, West China Hospital of Sichuan University, Guoxuexiang, No.37, Chengdu, 610041 China; 4 Department of Pathology, West China Hospital of Sichuan University, Guoxuexiang, No.37, Chengdu, 610041 China

**Keywords:** Paragonimiasis, Hepatic paragonimiasis, Diagnosis; infant

## Abstract

**Background:**

Paragonimiasis, particularly hepatic paragonimiasis (HP), is a type of zoonotic parasitic disease rarely encountered in infants. There have been only a few reports of HP, and no case of HP has been reported in an infant.

**Case presentation:**

A 15-month-old girl presented with persistent mild fever with a duration of 1 month, hepatomegaly, and low-density lesions in the right hepatic lobe on abdominal ultrasound and computer tomography. Pathological examination and serum antibody detection were performed to verify HP. The diagnosis of HP was established based on findings of Charcot-Leyden crystals on liver lesion biopsy and antibodies against *paragonimus westermani* detected by enzyme-linked immunosorbent assay. After initiation of praziquantel (75 mg/kg/day for 3 days), all clinical findings promptly improved and the patient was discharged.

**Conclusion:**

It is very important to consider paragonimiasis in the clinical examination of infants from an area with paragonimiasis epidemic presenting with fever, hepatomegaly, low-density lesions in the liver.

## Background

Paragonimiasis is a type of zoonotic parasitic disease caused by *Paragonimus westermani* (commonly referred to as the lung fluke) that has a wide distribution throughout China, Japan, Korea, Southeast Asia, and the Indian subcontinent [1–4]. In China, the nationwide prevalence is estimated at 1.7% [5]. Immature forms of *P. westermani* migrate through the duodenal wall, peritoneal cavity, and diaphragm, where they encapsulate and mature within the pulmonary parenchyma, with the majority of cases presenting with lung involvement [6]. Ectopic infections can occur in unexpected regions, including the liver [7], brain [8], muscles, and subcutaneous tissues [3]. The diagnosis of paragonimiasis is difficult owing to non-specific clinical symptoms, particularly in children, who cannot accurately express their symptoms and dietary history. Thus, a healthy eating habit is helpful for the prevention of paragonimiasis. Although there are only a few reports of hepatic paragonimiasis (HP) in adults [9], no report of HP in infants has been published to date. Liver biopsy is usually performed to diagnose HP or distinguish HP and other liver lesions [7]. Ultrasound, computed tomography (CT), and serum laboratory studies could be used to establish the diagnosis of HP, in addition to more invasive methods. Thus, it is very important to consider the possibility of this diagnosis during the clinical examinations of infants.

## Case presentation

**Fig. 1:**
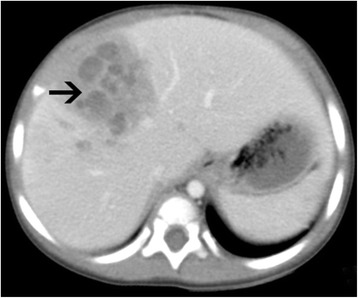
Computed tomography. Multiple low-density lesions in the right hepatic lobe (arrow),poorly circumscribed, with inhomogeneous enhancement, low-density necrosis, formation of visible partitions, and absent invasion of adjacent vessels

**Fig. 2:**
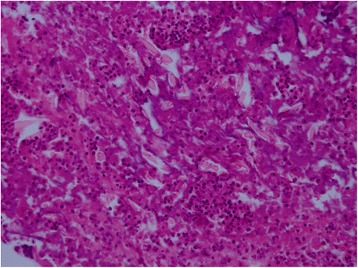
Photomicrograph of the liver surgical specimen. Charcot-Leyden crystals and numerous eosinophil scan be seen (hematoxylin and eosin stain, ×400)

A 15-month-old girl presented with persistent mild fever of 1-month duration, without cough, tachypnea, night sweats, weight loss, abdominal pain, or diarrhea. The patient exhibited no improvement after antibiotic treatment. On admission, no abnormality was identified on physical examination, with the exception of hepatomegaly up to 4 cm below the subcostal margin. Abdominal ultrasound was performed and showed a poorly defined, circumscribed, inhomogeneous echoic lesion with poor blood flow signals in the right hepatic lobe. Further abdominal CT (Fig. 1) showed multiple low-density lesions in the right hepatic lobe (arrow) (which was poorly circumscribed with inhomogeneous enhancement, suggestive of low-density necrosis), formation of visible partitions, and absent invasion of adjacent vessels. Hepatapostema was suspected, and liver biopsy was performed. Charcot-Leyden crystals were identified on microscopic examination (Fig. 2), raising the suspicion of HP.

The patient was from an area in Sichuan endemic for paragonimiasis, but her intake of freshwater crabs or crayfish could not be confirmed. Serum evaluation was positive for *P. westermani* antibodies, based on enzyme-linked immunosorbent assay (ELISA; which was performed by the Sichuan Province Center for Disease Control and Prevention). After the initiation of praziquantel treatment (75 mg/kg/day for 3 days), all clinical findings promptly improved and the patient was discharged.

## Discussion and conclusion

The low prevalence, non-specific symptoms, and low rate of detection of polypides or eggs make it difficult to diagnose HP, especially in children. The diagnosis is usually clinically based, without pathogen confirmation [10]. Elevated peripheral eosinophilia, history of consumption of freshwater crabs or crayfish, residence in a region with paragonimiasis epidemic, and/or serologic tests and imaging are the key in diagnosing paragonimiasis [10].

The symptoms of HP are usually variable and non-specific, including chronic right-upper-quadrant abdominal pain or back pain [7]. Some patients may remain asymptomatic until HP is discovered incidentally [7]. Therefore, the diagnosis of HP is difficult, especially in young children who cannot accurately express their symptoms. In the case reported here, our 15-month-old patient presented only with persistent fever and an increased CRP level; infection was suspected due to the elevated CRP and marked peripheral blood eosinophilia.

A definitive diagnosis of paragonimiasis is based on the presence of eggs in patient’s feces, sputum, or flukes in histological specimens. Eggs and polypides, however, cannot be detected in most lesions. Diagnosis can be confirmed by histopathological examination of biopsy tissue [10]. In our patient, repeated stool examinations to evaluate for presence of eggs showed negative results, and liver lesion biopsy revealed eosinophilic abscesses and Charcot-Leyden crystals, which is suspicious for paragonimiasis. However, obtaining tissue for histopathological evaluation is invasive, especially in the abdominal cavity. Thus, if HP could be correctly diagnosed by non-invasive examination methods, such as ultrasound or CT, the need for surgery can be reduced or even eliminated [9].

HP lesions are often incidentally detected by routine ultrasonography. Typically, HP shows subcapsular echoic lesions with irregular, tract-like, non-enhancing necrosis on contrast-enhanced ultrasound [1]. Based on the presence of liver enlargement, abdominal ultrasound and abdominal CT were performed in our patient, which showed inhomogeneous echoic lesions (Fig. 1) in the right lobe, which were suspected to be hepatapostema. However, the typical CT findings of HP are peripherally distributed lesions, mutually connected cysts with tortuous tract formation, or tubular enhancement, features that may be associated with the route of infection and migration of the worm [9];thus, HP may be confused with pyogenic abscess, hepatic tuberculosis, or cancer. A retrospective study was performed on 21 patients with HP, all of whom underwent surgery and were confirmed to have HP through surgical and histopathological identification [7]. In our case, liver biopsy was used to differentiate HP from cancer.

Another common method for confirmation of paragonimiasis is ELISA testing for serological diagnosis [11]. However, ELISA is used based on clinical suspicion, which would require the presence of markers such as eosinophilia, a known history of consumption of freshwater crabs or crayfish, or an area with paragonimiasis epidemic. In this case, ELISA was not performed before surgery because of the patient’s age, dietary history, and clinical symptoms.

We present this case to demonstrate that regardless of age or history of ingestion of freshwater crabs or crayfish, when ultrasound and/or CT show an inhomogeneous echoic lesion in the hepatic lobe, ELISA should be performed to confirm HP. This report should remind physicians that infants are also susceptible to paragonimiasis, and thus, it is very important to consider this diagnosis in the clinical examination of infants from an area with paragonimiasis epidemic presenting with fever, hepatomegaly, low-density lesions in the liver.

## Abbreviations

CRP: C-reactive protein
CT: computer tomography
ELISA: enzyme-linked immunosorbent assay
HP: hepatic paragonimiasis
